# Craniofacial Arteriovenous Malformation Diagnosed in the Neonatal Period: A Case Report

**DOI:** 10.7759/cureus.117486

**Published:** 2026-10-05

**Authors:** Mohammed A Obeid, Omran A Saeed, Naeem Alkheamy, Abedalkhalk A Khedr, Osama A Ali Yousif

**Affiliations:** 1 Education Center, Hamad Medical Corporation, Doha, QAT; 2 Medicine and Health Sciences, UCSI University, Kuala Lumpur, MYS; 3 Medical Education, Hamad Medical Corporation, Doha, QAT; 4 NICU, Hamad Medical Corporation, Doha, QAT; 5 Education, Hamad Medical Corporation, Doha, QAT

**Keywords:** arteriovenous malformations, congenital scalp avm, craniofacial avm, neonatal mri, neonatal vascular anomaly

## Abstract

Congenital arteriovenous malformations (AVMs) in neonates present a significant diagnostic challenge due to their clinical overlap with benign vascular lesions such as infantile hemangiomas, requiring detailed clinical and radiological evaluation to exclude high-flow dynamics, muscular invasion, or intracranial extension.

A term male neonate presented with a localized right temporo-facial hemangioma-like lesion. Bedside cranial and abdominal ultrasonography excluded visceral anomalies and intracranial pathology. Contrast-enhanced Head MRI confirmed a high-flow right temporal AVM involving the temporalis muscle and extending to the zygomatic/orbital region, predominantly supplied by external carotid artery branches without major middle cerebral artery involvement.

The patient was evaluated by an interdisciplinary team comprising neonatology, pediatric neurosurgery, and plastic surgery. After neurosurgical clearance, the infant was discharged home in stable condition on full feeds. At the post-discharge NICU follow-up clinic, the infant demonstrated excellent clinical progress, normal neurodevelopment, and appropriate physical growth with active feeding and stable vital signs. Referrals were established for subspecialized follow-up in Dermatology.

Neonatal vascular markers necessitate systematic multi-modal imaging to define anatomical extent and vascular dynamics. Early multidisciplinary collaboration enables safe conservative management, neurosurgical clearance, and structured outpatient surveillance across pediatric surgical and dermatological specialties.

## Introduction

Vascular anomalies are broadly divided into vascular tumors and vascular malformations, both of which may have overlapping clinical features, making accurate diagnosis challenging, especially in the neonatal period. The International Society for the Study of Vascular Anomalies (ISSVA) classification distinguishes vascular tumors from vascular malformations according to their biological and clinical characteristics. Arteriovenous malformations (AVMs) are fast-flow vascular malformations characterized by multiple direct communications between arteries and veins, with enlargement of feeding and draining vessels. Although AVMs may become clinically apparent later in life, approximately one-third are evident at birth [1,2].

Congenital scalp AVMs are particularly rare lesions that can involve the scalp, face, and deeper adjacent structures. Their clinical appearance may be variable, ranging from subtle cutaneous discoloration to more prominent pulsatile or mass-like lesions. In neonates, congenital scalp AVMs are particularly rare and may be mistaken clinically for hemangiomas or other vascular lesions. Published neonatal cases have described temporal or frontotemporal AVMs initially considered to represent hemangioma, highlighting the difficulty of establishing the diagnosis based on clinical appearance alone [3,4].

Therefore, imaging plays an important role when clinical features are insufficient for diagnosis. Its use is particularly important when the lesion has atypical features or involves anatomically critical regions. Doppler ultrasonography and magnetic resonance imaging can help characterize flow dynamics, define the extent of the lesion, and identify arterial feeders and venous drainage. MRI may demonstrate features of a high-flow lesion, including prominent vascular channels and flow voids, while defining involvement of adjacent soft tissues and intracranial or orbital structures. Accurate characterization is important because vascular tumors and malformations differ substantially in their natural history and management [5,6].

We report a term neonate with a right temporal vascular lesion identified shortly after birth and initially suspected to represent a hemangioma. MRI demonstrated features consistent with a high-flow vascular malformation with arterial supply from branches of the external carotid artery and extension toward the orbit and temporalis muscle, leading to a diagnosis of a craniofacial AVM.

## Case presentation

A term male infant was born at 40 weeks and 3 days' gestation to a gravida 1, para 1 mother following an uncomplicated spontaneous vaginal delivery with difficult extraction. His Apgar scores were reassuring at 9 and 10 at one and five minutes, respectively, and his birth weight was 3.742 kg. The pregnancy was uncomplicated; however, antenatal ultrasound was limited by poor visualization of the fetal face, and the lesion was therefore not identified prospectively. Maternal GBS screening was negative, and there was no significant family history.

The anomaly was identified in the immediate aftermath of delivery as a vascular-appearing lesion over the right temple (Figure 1). Initially, it was thought to represent a hemangioma. Given its proximity to the eye and ear, the decision was made to admit the infant to the NICU for further evaluation rather than discharge him home.

**Figure 1:**
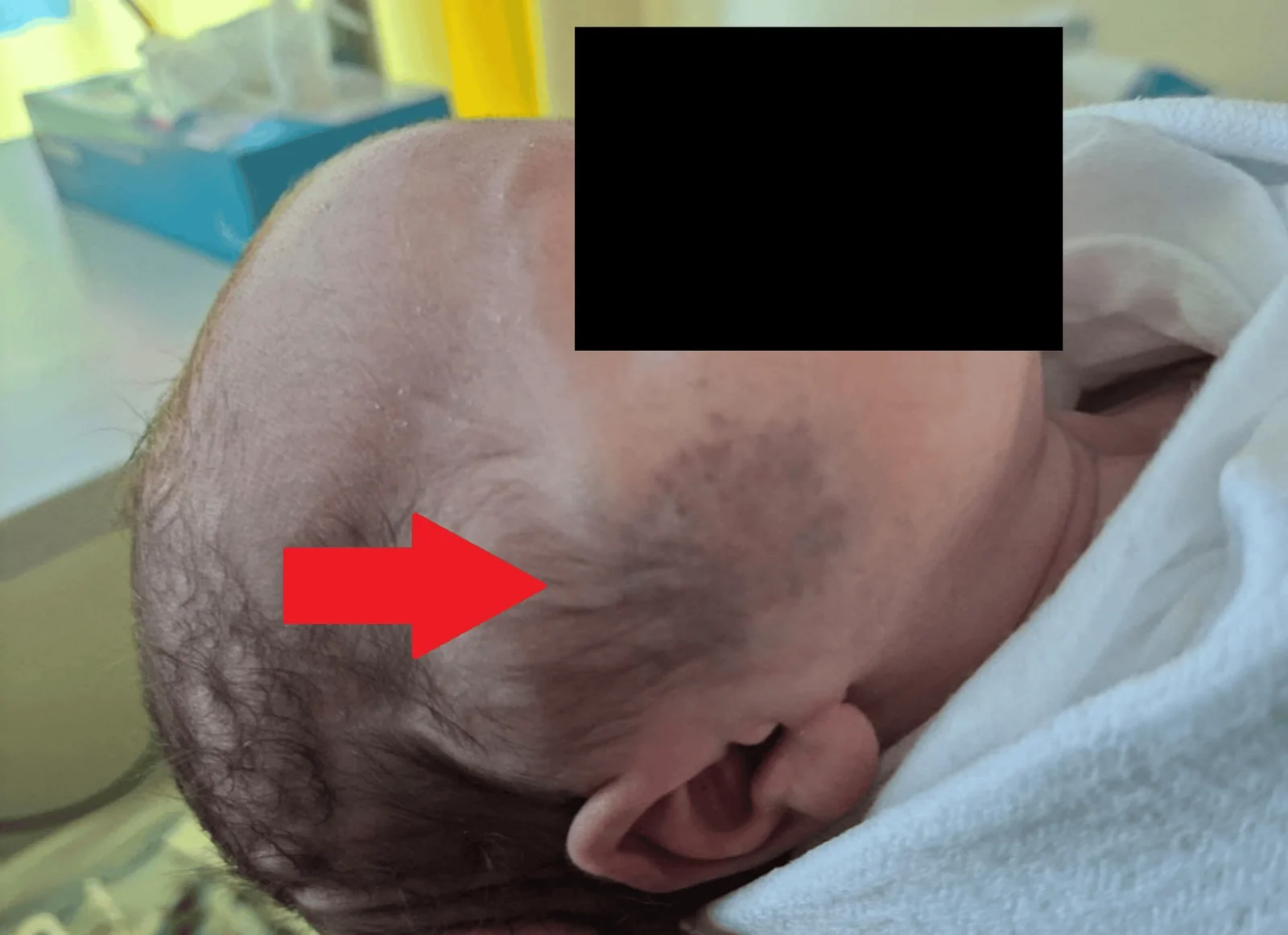
Clinical photograph taken shortly after birth showing a flat, dusky violaceous discoloration over the right temporal and periauricular scalp, extending toward the right forehead. The lesion is macular to minimally raised, with no ulceration, bleeding, or crusting, and gradually fades into the surrounding hair-bearing scalp. The remainder of the infant's skin and coloration were unremarkable. The red arrow indicates the arteriovenous malformation.

On the first day of life, the infant was doing well, active, pink, and feeding fully, with reassuring examination findings throughout. The anterior fontanelle was soft and flat, with no jaundice; heart sounds were normal, the chest was clear, the abdomen was soft, and the neurological examination revealed no concerns. His vital signs remained stable throughout (temperature, 36.8°C; heart rate, 144 bpm; respiratory rate, 44 breaths/min; blood pressure, 77/43 mmHg; oxygen saturation, 97% on room air), and he was tolerating breastfeeding. The patient remained hemodynamically stable, and bedside ultrasonography of the abdomen and pelvis was normal.

Given the lesion's proximity to the orbit and its somewhat atypical appearance on examination, neurosurgery was consulted early, and an MRI of the brain and head was arranged.

MRI of the head

In summary, MRI performed to rule out an intracranial abnormality in a patient with a presumed hemangioma instead identified a 3.7 × 1.7 × 3.4 cm right temporal soft-tissue lesion extending to the zygomatic region and posterior orbital wall. The lesion demonstrated mixed, predominantly high T2 signal, with linear branching areas and signal voids consistent with flow. It was subcutaneous, involved and mildly expanded the right temporalis muscle, and was supplied primarily by branches of the right external carotid artery, with only a possible small intracranial perforator and no major right MCA supply. The brain parenchyma was normal, with no midline shift, mass effect, restricted diffusion, susceptibility abnormality, hemorrhage, or extra-axial collection, and the ventricles were normal in size (Figure 2). Taken together, these findings pointed away from a simple hemangioma and toward an AVM.

**Figure 2:**
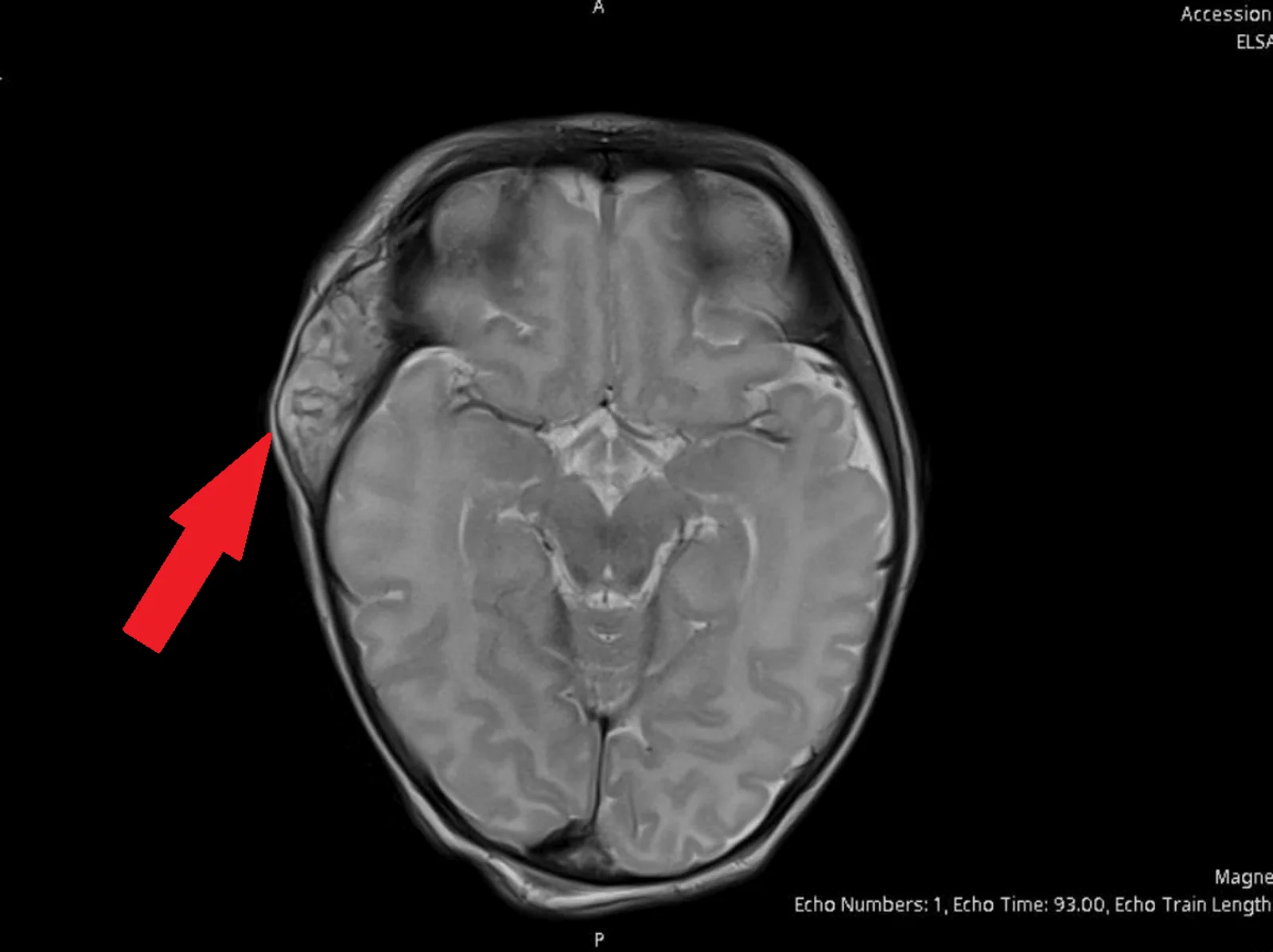
Axial T2-weighted MRI of the brain (t2_qtse_tra sequence) showing normal brain parenchyma with no midline shift, hemorrhage, or extra-axial collection. The ventricles are normal in size and configuration. The red arrow indicates the arteriovenous malformation.

MR angiography (coronal maximum-intensity-projection reconstruction) demonstrated an abnormal, irregular tangle of vessels over the right temporal region, with feeding branches from the external carotid circulation, consistent with an AVM (Figure 3).

**Figure 3:**
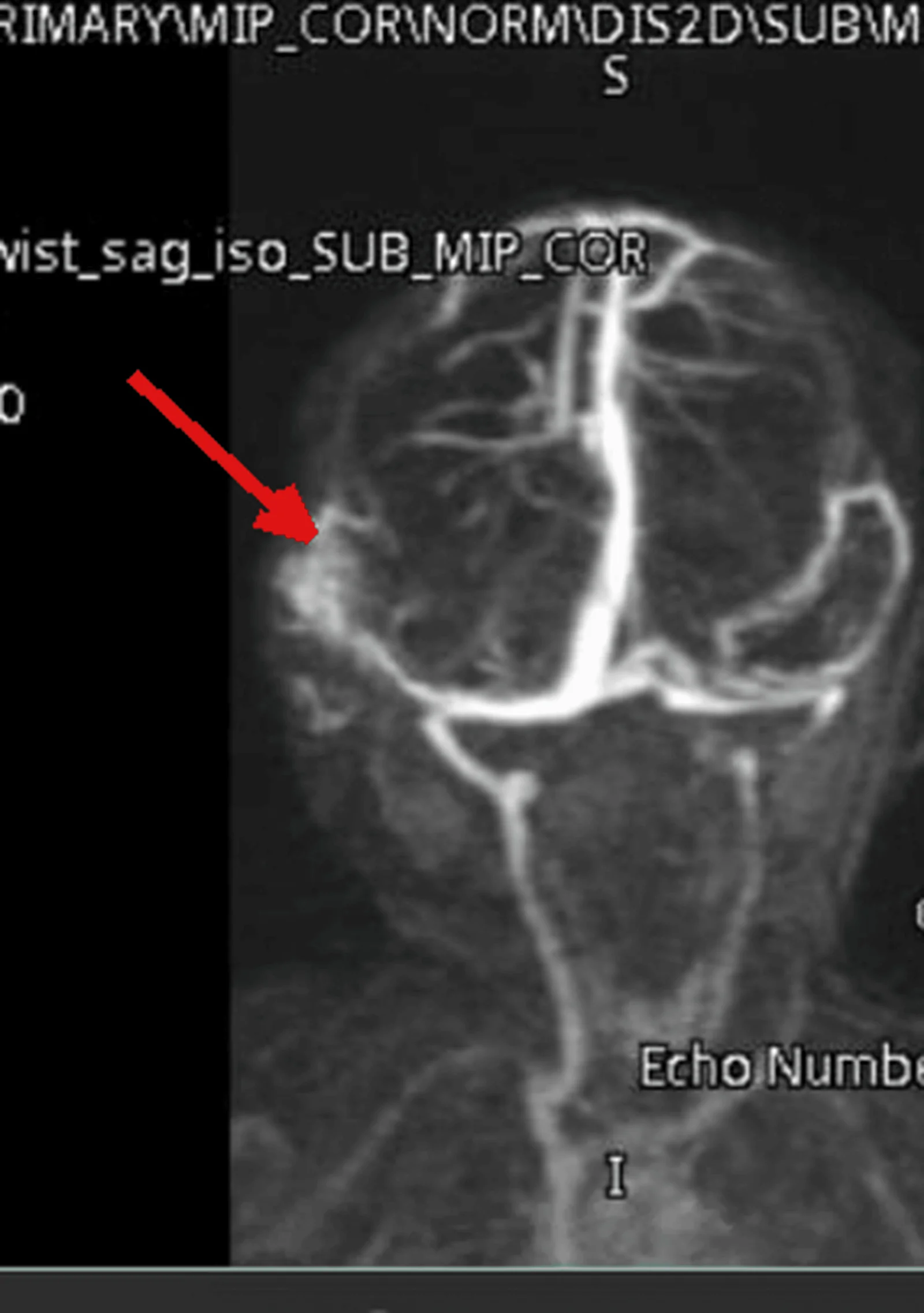
Coronal maximum-intensity-projection (MIP) MR angiogram of the head showing an abnormal, irregular tangle of vessels over the right temporal region (arrow), consistent with an arteriovenous malformation with feeding branches from the external carotid circulation.

Taken together, the flow voids, the external carotid supply, and the muscular involvement indicated that this lesion demonstrated features exceeding the typical diagnostic criteria for a simple hemangioma. After discussion between neonatology, neurosurgery, and plastic surgery, the consensus was that this represented a craniofacial AVM.

By day of life 4, he was doing well from every other standpoint - active, pink, feeding fully, with reassuring examination findings throughout: soft anterior fontanelle, no jaundice, normal heart sounds, clear chest, soft abdomen, and a neurological examination that raised no concerns whatsoever. His vital signs were stable throughout (temperature, 37.0 °C; heart rate, 145 bpm; respiratory rate, 57 breaths/minute; blood pressure, 75/39 mmHg; oxygen saturation, 99% on room air), and he was tolerating around 120 mL/kg/day of feeds with normal urine output and stooling. The patient had no signs or symptoms suggestive of sepsis or systemic illness.

Routine bloodwork was reassuring, with all laboratory investigations within the reference range. His newborn metabolic screen - including 17-hydroxyprogesterone, amino acid/acylcarnitine profile, and biotinidase - came back clear, as did G6PD screening. There was no evidence of systemic involvement, and the findings were consistent with a localized lesion; it appeared to be a local, isolated problem. Because he remained clinically well throughout - with no neurological signs, no evidence that the lesion was stealing blood flow or causing any cardiovascular strain, and no concerning intracranial extension - the team felt there was no indication for urgent intervention during this admission. He continued to do well and was discharged.

Rather than intervening acutely, the plan was to refer the infant to the vascular anomalies clinic (JVAC), a multidisciplinary service with expertise in the longitudinal management of these lesions. Two weeks later, at the post-discharge NICU follow-up clinic, the infant showed strong clinical progress, normal neurodevelopment, and healthy physical growth, marked by active feeding and stable vital signs. Subspecialized outpatient follow-up was arranged with Dermatology.

## Discussion

AVMs are characterized by a direct connection between the arteries and veins, bypassing the normal capillary bed, with blood shunting directly from the arterial to the venous side. They occur predominantly in the cranial cavity but can be present outside the cranium, including in the scalp and face, as in the case of our patient. Such extracranial AVMs are usually congenital and do not manifest with symptoms until the child reaches adolescence or adulthood, when symptoms such as bleeding, pulsation, or enlargement of the lesion may develop [3]. Our patient represents the rare occurrence of the diagnosis being made soon after birth, before the appearance of these symptoms [7].

Although the etiology of AVMs has yet to be fully elucidated, it is known to include congenital developmental abnormalities of blood vessel formation, combined with genetic, hormonal, and biochemical factors. Increased expression of the vascular endothelial growth factor (VEGF) protein in the affected areas has also been found in some studies.

AVMs can be distinguished from hemangiomas by their underlying biology. Hemangiomas are proliferative vascular tumors, whereas vascular malformations, including AVMs, result from congenital abnormalities of blood vessel development.

The distinction is made by the ISSVA classification [8]. One helpful diagnostic feature to consider in this context is the lesion's growth pattern: a hemangioma that appears at birth with clear margins, without any further growth, is likely to be a congenital hemangioma, while aggressive and vertical growth within the first six months of life suggests an infantile hemangioma. AVMs, on the other hand, do not meet the above criteria - they can be superficial and present early in life, but can also be deep and not discovered until much later in life, because deep AVMs are asymptomatic for many years despite the vascular malformation being already developed at birth. Since AVMs infiltrate the normal soft tissues around them rather than create a tumor, the management of AVMs is fundamentally different from that of hemangiomas; the former requires staged multidisciplinary treatment, including lasers, embolization, and other selective procedures to preserve the normal architecture, while the latter can be treated medically (e.g., propranolol) (Table 1).

**Table 1:** Comparison of infantile hemangioma, congenital hemangioma, and arteriovenous malformation. RICH, rapidly involuting congenital hemangioma; NICH, non-involuting congenital hemangioma; PICH, partially involuting congenital hemangioma; MRA, magnetic resonance angiography; ISSVA, International Society for the Study of Vascular Anomalies; MRI, magnetic resonance imaging; GLUT1, glucose transporter 1; MRA, magnetic resonance angiography

Feature	Infantile hemangioma	Congenital hemangioma (RICH/NICH/PICH)	Arteriovenous malformation
ISSVA category	Benign vascular tumor	Benign vascular tumor	Fast-flow vascular malformation
Timing of onset	Absent or minimal at birth; appears in the first weeks of life	Fully formed at birth	Present at birth (may be quiescent or subtle); may become apparent later
Natural history	Rapid proliferation in the first 3-6 months, then slow involution over years.	RICH: involutes rapidly, usually within the first 12-14 months. NICH: does not involute. PICH: partial involution	No spontaneous regression; grows with the child and may expand with puberty, hormonal change, or trauma
Typical appearance	Red or bluish plaque or nodule; deep lesions appear as a bluish swelling	Round or oval, raised, violaceous, often with a pale halo (RICH) or a flat, pink to bluish plaque (NICH)	Pink-red or violaceous stain or swelling; warm, with a palpable thrill or pulsation in advanced stages
Doppler ultrasound	Hypervascular soft-tissue mass with high vessel density	High-flow, localized soft-tissue mass; RICH may mimic a shunt	High-flow arterial and venous signals without a discrete mass; dilated feeding and draining vessels
MRI/angiography	Well-defined lobulated mass, intermediate T1 and high T2 signal, avid enhancement	Well-defined mass; internal flow voids may be present	Ill-defined, infiltrating lesion with flow voids and no discrete mass; MRA or catheter angiography shows arterial feeders and early venous filling
Pathology	GLUT1-positive	GLUT1-negative	Abnormal arteriovenous channels with no capillary bed; no endothelial proliferation
Evaluation	Usually clinical; ultrasound or MRI for deep, segmental, or high-risk sites	Clinical and Doppler ultrasound; blood count and coagulation profile in large RICH	Doppler ultrasound and MRI/MRA; angiography to plan treatment; clinical staging
Treatment	Observation for uncomplicated lesions; propranolol first line for problematic or high-risk lesions; topical timolol for small superficial lesions	RICH and PICH: observation. NICH: observation or excision if symptomatic or for cosmetic reasons	Observation if asymptomatic; embolization with or without surgical resection if symptomatic; laser for cutaneous component; targeted agents under study

Kasabach-Merritt phenomenon

Kasabach-Merritt phenomenon (KMP) is a consumptive coagulopathy with profound thrombocytopenia, low fibrinogen, and raised fibrin degradation products. It is a life-threatening complication that is important to recognize in neonates with a vascular lesion, but it is associated with the vascular tumors kaposiform hemangioendothelioma and tufted angioma, not with AVMs or typical infantile hemangiomas [9,10]. These lesions are usually firm, rapidly enlarging, and purpuric or violaceous. Our patient's lesion was flat to minimally raised; he had no bleeding, and his platelet count and coagulation profile were normal, so KMP was excluded. Platelet count and coagulation profile should be checked in any neonate with a rapidly growing or firm violaceous vascular lesion.

Symptomatic neonatal scalp AVMs are exceedingly rare. Hussain et al. described a symptomatic scalp AVM in a 10-day-old neonate, noting that such lesions are often misdiagnosed as hemangiomas and that only two symptomatic neonatal scalp AVMs had been reported before their case [3]. In contrast, our patient was asymptomatic at presentation, with a flat violaceous discoloration and no bleeding, ulceration, or crusting, and the lesion was investigated because of its location near the eye and ear rather than because of complications. Abreu et al., in a case report and literature review of neonatal AVMs, likewise emphasized that incorrect nomenclature and misdiagnosis are frequent and that AVMs are potentially the more aggressive type of vascular malformation [4]. Taken together with our case, these reports support a low threshold for imaging any neonatal vascular lesion that has atypical features or lies in a high-risk location.

The overlap between high-flow vascular tumors and malformations in the neonatal scalp is further illustrated by lesions labelled as congenital hemangiomas that behaved as high-flow shunts. Shah et al. reported a 29-week premature infant with a suspected large parietal scalp hemangioma in whom bedside Doppler ultrasound showed arteriovenous shunting and who had high-output cardiac failure treated with endovascular embolization [11]. Alluhaybi et al. similarly described a premature newborn with a giant scalp congenital hemangioma and cardiac failure who underwent embolization of the superficial temporal artery followed by surgical excision [12]. These cases show that high-flow scalp lesions in neonates can be hemodynamically significant. Our patient, by contrast, had no cardiac or neurological compromise, which allowed conservative management with neurosurgical clearance and outpatient surveillance.

Imaging was central to the diagnosis in our patient. MRI is regarded as the most valuable modality for classifying vascular anomalies because it demonstrates the extent of a lesion and its relationship to adjacent structures, and dynamic time-resolved contrast-enhanced MR angiography can differentiate high-flow from low-flow lesions [5,13]. In our patient, the flow voids, external carotid supply, and temporalis muscle involvement were features of a high-flow lesion and argued against a simple hemangioma, while the normal brain parenchyma and absence of a major MCA contribution supported a lesion confined to the extracranial soft tissues.

Scalp AVMs more commonly present later in life. Talafha et al. reported a 17-year-old with an enlarging, pulsatile parietal scalp mass and mild headaches, treated successfully with endovascular transarterial embolization, and highlighted the importance of digital subtraction angiography for defining the angioarchitecture and planning management [14]. Such reports illustrate the course our patient may follow, since clinical manifestations of scalp AVMs may emerge in adolescence or adulthood or after trauma [3], and the treatment options available should the lesion become symptomatic [8,14]. For now, our patient is being managed conservatively with structured follow-up because he had no signs of hemodynamic compromise or intracranial extension.

## Conclusions

Superficial facial vascular lesions in neonates require careful evaluation, as benign-appearing hemangiomas can clinically mask underlying high-flow AVMs. Bedside ultrasonography provides an effective initial screen to detect intracranial or visceral extension, while contrast-enhanced MRI remains essential for definitive anatomical and vascular mapping. Multidisciplinary consultation enables safe neurosurgical clearance and conservative management, supported by outpatient dermatology follow-up.
